# Low-field Switching Four-state Nonvolatile Memory Based on Multiferroic Tunnel Junctions

**DOI:** 10.1038/srep12826

**Published:** 2015-08-04

**Authors:** H. M. Yau, Z. B. Yan, N. Y. Chan, K. Au, C. M. Wong, C. W. Leung, F.Y. Zhang, X. S. Gao, J. Y. Dai

**Affiliations:** 1 Department of Applied Physics, The Hong Kong Polytechnic University, Hung Hom, Kowloon, Hong Kong, P. R. China; 2 Laboratory of Solid State Microstructures, Nanjing University, Nanjing 210093, China; 3 Institute for Advanced Materials and Laboratory of Quantum Engineering and Quantum Materials, South China Normal University, Guangzhou 510006, China

## Abstract

Multiferroic tunneling junction based four-state non-volatile memories are very promising for future memory industry since this kind of memories hold the advantages of not only the higher density by scaling down memory cell but also the function of magnetically written and electrically reading. In this work, we demonstrate a success of this four-state memory in a material system of NiFe/BaTiO_3_/La_0.7_Sr_0.3_MnO_3_ with improved memory characteristics such as lower switching field and larger tunneling magnetoresistance (TMR). Ferroelectric switching induced resistive change memory with OFF/ON ratio of 16 and 0.3% TMR effect have been achieved in this multiferroic tunneling structure.

Resistance switching is one of the major mechanisms for non-volatile memories, and resistive switching memory based on metal-insulator-metal (MIM) structure has been extensively studied for more than a decade. In resistive switching process, with a high voltage applied, the insulating film can be made to be conductive through a filament conduction path which is usually formed by defects and metal migration inside the insulator or near the metal/insulator interface. However, resistive switching is still a destructive process because of the defect and distortion in the insulating layer after applying a sufficient high voltage. In order to improve the memory device performance, many memory structures are being studied according to the properties and features of materials. One of the hottest topics in this field nowadays is ferroelectric switching, from which the resistance switching can be obtained without forming any filament, and it has a much faster response time as well. The unique polarization switching in ferroelectrics has been found to affect electron transport properties significantly, and it has been demonstrated that such a tunnel junction can achieve giant electroresistive effect in which the resistance changes as high as 2–3 orders accompanied by a ferroelectric polarization switching^1^,^2^. This may lead to a sort of simple architecture of nonvolatile random access memory (RAM) that can overcome the storage density limitation of current devices. In addition, as the magnetic materials are used in the two conducting metal layers, a multiferroic tunnel junction can be realized, in which ferroelectric-ferromagnetic coupling and their interplay allow electrical control of magnetism or the extent of spin polarized electrons. This presents a way for a type of reversible, non-volatile, and potentially low-power four-state logic devices.

Aiming to fabricate a ferroelectric tunnel junction, an ultrathin ferroelectric layer is needed as a barrier for tunneling. Contreras *et al.* has experimentally investigated an ultrathin barrier in metal-ferroelectric-metal junction using Pb(Zr_0.52_Ti_0.48_)O_3_ and obtained a switching effect with resistance OFF/ON ratio close to 4 due to a pure ferroelectric polarization^3^. Maksymovych *et al.*^4^ demonstrated a highly reproducible control of electric transport by spontaneous polarization of a ferroelectric oxide Pb(Zr_0.2_Ti_0.8_)O_3_, where the hysteretic tunneling current coincides with ferroelectric polarization. Many researches^1^,^3^,^5^,^6^,^7^,^8^,^9^,^10^ have provided evidence for the correlation between the ferroelectric polarization switching and resistance switching.

In this work, BaTiO_3_ (BTO) is used as the ultrathin ferroelectric barrier sandwiched by two electrodes composed of different magnetic materials La_0.7_Sr_0.3_MnO_3_ (LSMO) and Permalloy Ni_0.81_Fe_0.19_ (NiFe), which are chosen as bottom and top electrode respectively. As a half-metallic ferromagnet, the Curie temperature of LSMO is higher than room temperature, and in particular its spin polarization is close to 100%^11^. On the other hand, NiFe is chosen as the top electrode because it is a well-known alloy that allows strong spin electron injection at room temperature, and has relatively much lower coercive field, which makes it able to form a low switching field spin valve structure.

## Results and Discussion

**Figure 1:**
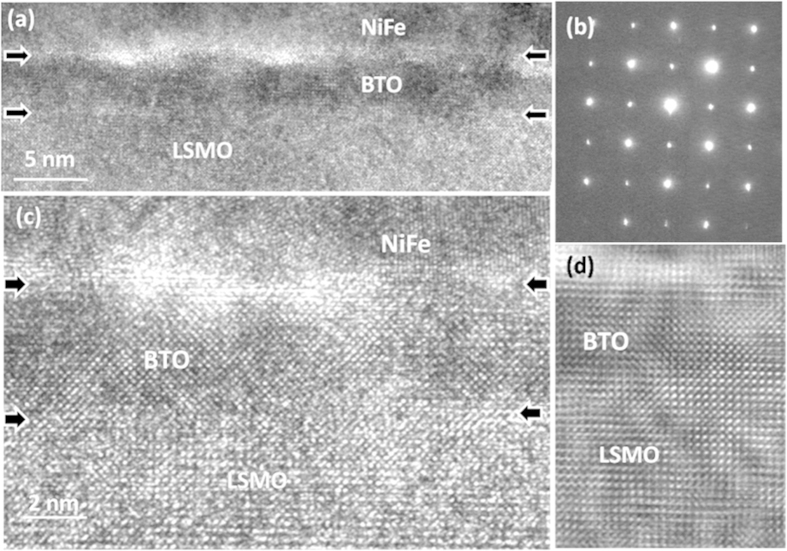
Structure of NiFe/BTO/LSMO/STO heterostructure: (**a**) Low magnification TEM cross-section image of 5 nm-BTO on LSMO grown on STO substrate; (**b**) the corresponding electron diffraction pattern including the STO substrate and BTO/LSMO layers; (**c**) High-resolution TEM image of the NiFe/BTO/LSMO observed along [100] direction; and (**d**) a noise-reduced image from (c).

A 5nm-thick BTO ultrathin film was deposited epitaxially on 60-nm thick LSMO film which is confirmed as metallic behavior acting as bottom electrode with a good performance of transport properties (Supplementary Figure S1). Figure 1(a) shows a low-magnification cross-sectional TEM image of the NiFe/BTO/LSMO film on STO substrate, where a continuous layer of about 5 nm BTO can be clearly seen and the epitaxial growth of BTO on LSMO with sharp interface can be confirmed (also illustrated by selected area electron diffraction in Fig. 1(b)). High-resolution TEM image of the BTO film on LSMO layer, as shown in Fig. 1(c), reveals the excellent epitaxial growth of BTO on LSMO; the continuity growth of BTO on LSMO makes their interface hard to be distinguished as can be seen in the noise-reduced image as shown in Fig. 1 (d). Since the BTO has relatively larger lattice constants (a = 3.992 Å, c = 4.036 Å) compared to that of LSMO and STO, such a stacking structure can also ensure that the BTO is under a compressive stress with c-axis along the film normal direction. This is very important for ferroelectric switching memory application^12^.

**Figure 2:**
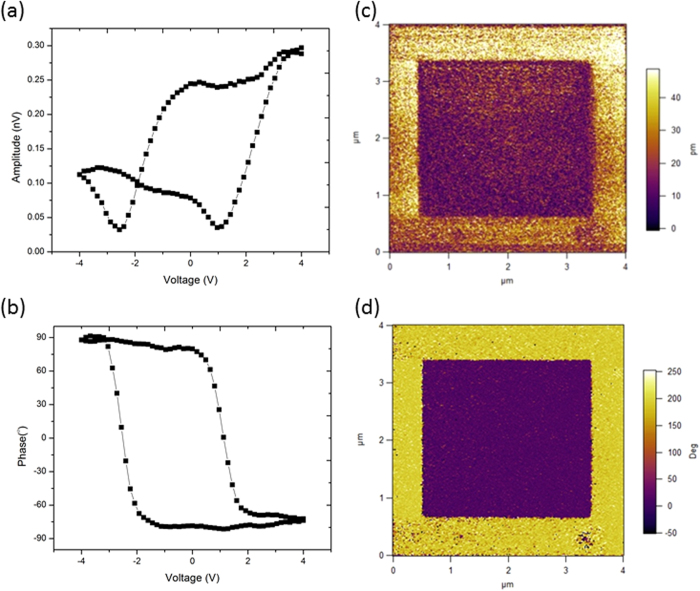
Ferroelectricity of BTO Ultrathin film. (**a**) and (**b**) butterfly loop amplitude and phase curves of the 5 nm-BTO film; PFM out-of-plane amplitude (**c**) and phase (**d**) images recorded after writing an area of 1 μm x 1 μm with −2.5 V and then the central square with +2.5 V using a biased conductive tip.

To prove the ferroelectric property of the ultrathin BTO film, which is essential for this multiferroic tunneling junction, piezoresponse force microscopy (PFM) was used to demonstrate BTO’s ferroelectric switching. Figure 2(a) are the butterfly loop amplitude and phase curves of the device, where one can see that the butterfly loop is asymmetric, and we believe that it should be caused by the different work functions of LSMO and NiFe. The out-of-plane PFM amplitude and phase images are shown in Fig. 2(c) recorded as a ferroelectric domain structure written on the BTO surface with ± 2.5 V. The 180° phase contrast reveals the domain structure showing two antiparallel polarizations written by poling voltage. Electrons accumulation and depletion at the interface of BTO and LSMO based on the explanation from Wen *et al.*^13^ may also occur in this structure. Due to the electron screening effect, different directions of polarization may change the band diagram and thus widen the tunneling barrier as well as the coercive voltage in different polarity.

**Figure 3:**
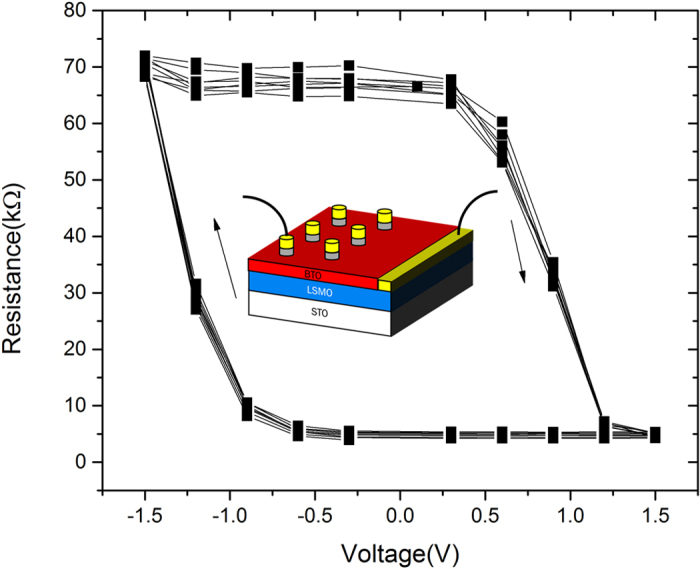
Stability of OFF/ON electroresistance change in the devices. Resistance-voltage curves obtained at 40 K for 10 cycles shows a stable performance of the device.

Resistance-voltage curves showing non-volatile resistance switching is demonstrated in Fig. 3, and the schematic diagram of the multiferroic tunneling junction is shown in the inset of the figure. During the measurements, pulse train writing voltages were used in the sequence of 0V→1.5 V→0V→–1.5 V→0 V with a step of 0.2 V, and the reading voltage remained unchanged as V_read_ = 0.1 V. At 40 K, this device exhibits a characteristic of bipolar resistive switching with an electroresistance OFF/ON ratio of 14. For this device, no electroforming at higher voltage is needed before the observation of hysteretic resistance-changes. A positive pulse of voltage drives the BTO polarization to point to NiFe which sets the device to low resistance state LRS (the top electrode is grounded and the voltage is applied on the bottom LSMO electrode). While to set the device to high resistance state HRS, a negative voltage pulse drives the polarization to the opposite. It should be noticed that at room temperature, there is only an extremely small OFF/ON ratio, i.e. the resistance-voltage (R-V) curve measured at room temperature is not significant enough compared with that measured at 40K. The reason that the low temperature measurement shows much larger OFF/ON ratio is due to the suppress of leakage current at low temperature; while at room temperature oxygen vacancy and other defect induced leakage current overrides the ferroelectric switching induced resistive switching (Supplementary Figure S2 and S3). Moreover, by taking account of the temperature-dependent resistance of LSMO, resistance of the bottom electrode exponentially decreases in lower temperatures. From Fig. 3, it can also be seen that after ten cycles, the resistance shows identical loops, indicating a good reproducibility of the device performance. Also, the HRS and LRS are stable and flatten unless it reaches the coercive voltage, which shows the stability of the device.

**Figure 4:**
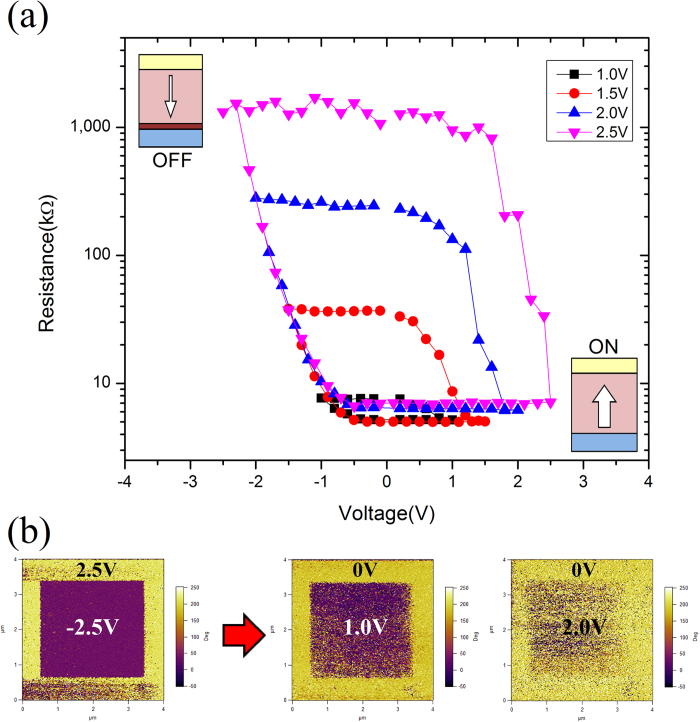
(**a**)Resistance hysteresis loops of FTJ measured at 40 K using the pulse voltage to write and changing with the maximum voltage increasing from 1.0 to 2.5 V were applied. Read pulses of 0.1 V were applied after each write pulse. The corresponding domain evolution is shown schematically in the bottom-right and top-left insets for the ON and OFF states, respectively. (**b**) PFM phase images of BTO areas switched with the voltage used in the measurement. Relative fraction of switching domains show states achieved by the application of positive (and negative) voltage pulses of increasing amplitude.

Figure 4(a) shows the resistance changes with different writing voltages at low temperature (40 K), where it can be seen that the device shows uniform performance. The R-V curves record the junction resistance measured at V_read_ = 0.1 V with varying maximum V_write_. One can see that at the negative voltage side, the coercive voltages for different writing voltage are the same, while they are different at the positive side. This is consistent with asymmetric butterfly loop shown in Fig. 2. This result is similar to André Chanthbouala’s work^14^, and the reason for this phenomenon is due to the fact that a larger writing voltage makes the polarization switching more complete, and therefore, the resistance shows larger OFF/ON ratio. This can be demonstrated in the Fig. 4(b), where it is apparent that the relatively lower voltages below 2.5 V result in partially switched domain structure. For Fig. 4(b), the change of upward and downward polarization in BTO film can also determine the tunneling electro-resistance effect (TER)^15^. Domain can be switched only when a tip is biased with a voltage that exceeds the coercive voltage for this film from the result of PFM butterfly loop. This suggests polarization switching of this ferroelectric is present during the switching. Also, conductance of HRS and LRS for our device is fitted by Brinkman’s model^16^ and Simmons’ model^17^ (Supplementary Figure S4 and S5) which also illustrates the TER effect in our FTJ device in low temperature. To explain the variation of OFF state resistance, BTO/LSMO interface screening effect^1^,^18^ should be considered. Besides the increase of barrier height when polarization is downwards pointing to the LSMO, the tunneling transmittance also reduced. Tsymbal *et al.*^19^ and Wen *et al.*^20^ have mentioned that accumulation and depletion of the majority carriers would occur at the semiconductor interface in the OFF state, and thus, an extra barrier against the tunneling electrons appears in the depletion region near to LSMO surface. By increasing the maximum voltage, the width of the depleted space charge region increases, and therefore, the curves with different V_max_ do not coincide in the OFF state.

After determining the electrical transport properties of our ferroelectric tunnel junction, magnetic properties of the LSMO and NiFe electrodes were examined. In spite of the model of Dieny *et al.*^21^, a modern spin valve structure of our device is made of two dissimilar ferromagnetic materials, LSMO and Ni_0.81_Fe_0.19_, as the electrodes. Because of two different coercive fields, soft magnetic material and hard magnetic material can be categorized and used for a pseudo spin valve due to the parallel and antiparallel configuration of magnetic properties. When the electrodes are ferromagnetic, electrons tunnel across the insulating barrier are spin-polarized, and this polarization reflects the density of states at the Fermi level of the electrodes. This spin polarization is the origin of the tunneling magnetoresistance (TMR). Several groups of researchers have reported about the further development of ferromagnetic devices – tunnel magnetoresistance^22^,^23^,^24^,^25^,^26^. To obtain the ratio of TMR, tunnel magnetoresistance can be expressed in equation (1) by using Julliere model^27^:
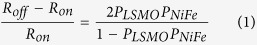
Where R_off_ is the resistance in antiparallel magnetization state, R_on_ is that in parallel magnetization state, P_LSMO_ and P_NiFe_ are the spin polarizations of the two electrodes. According to the above theory, when two ferromagnetic electrodes sandwiched with a thin layer of insulating material, a magnetic tunnel junction can be made. In our expectation, LSMO which had been tested as almost 100% positive spin-polarization and most of the ferromagnetic metals such as Co and Ni are negative^28^,^29^, so it can be assumed that our results of TMR would be negative.

**Figure 5:**
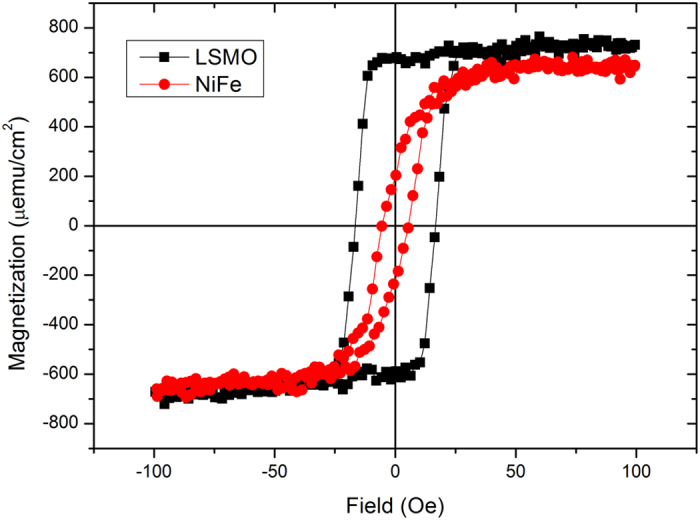
Magnetism of NiFe and LSMO. Hysteresis loop of NiFe and LSMO (at 8 K temperature).

To aim at TMR, double hysteresis loops should be obtained in two dissimilar electrodes. According to the moment-field loop of NiFe and LSMO obtained by VSM shown in Fig. 5, at low temperature (8 K), the coercive field of LSMO is 20 Oe, and it is 5.0 Oe for NiFe in the direction of parallel to film plane. By comparing with these two magnetic materials, the NiFe electrode should act as a soft magnet while the LSMO layer should act as a hard magnet. This result suggests that the so-called four-state memory function can be realized by coupling and interplay between the magnetic and ferroelectric polarizations at this multiferroic tunneling structure. The magnetization direction dependent-resistance for this tunneling junction can be explained by tunneling magnetoresistance (TMR) which is about spin-polarized tunneling that involves alignment of magnetic moments of the two magnetic electrode layers^22^,^29^. According to Kouacou’s research in 2008^30^, LSMO/STO(001) has an easy magnetization direction with [110] as the easy-axis while along the perpendicular axis, the saturation field is much higher (the saturation field μ_0_H_sat_ is found to be 7,000G at 285 K and 14,000G at 10 K), so there is no noticeable change in resistance because of the relatively much weaker magnetic field applied. Therefore, the loop is analogous to that measured without applying a magnetic field. However, when the magnetic field is parallel to the film plane, the relative magnetization directions of the two ferromagnetic layers are easily aligned in parallel due to the direction of easy axis, and therefore, very clear TMR effect can be observed.

**Figure 6:**
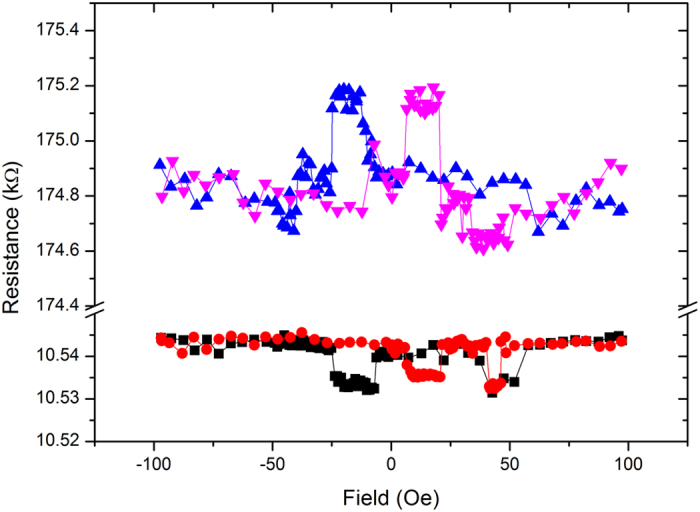
Resistance changes with different magnetic field at low temperature (8 K). It shows four-state memory tested on the NiFe/BTO/LSMO structure.

The four-state non-volatile memory characteristic of this multiferroic tunnel junction was further demonstrated by the magnetic and electrical switching measurement^29^,^31^,^32^,^33^,^34^. During measurement, a device of NiFe/BTO/LSMO was placed in the middle of two current-controlled electromagnets with the magnetic field parallel to the film direction aiming to magnetize the NiFe and LSMO electrodes to the same direction. By poling the ferroelectric barrier upwards and downwards, then switching magnetic field from 100 Oe to −100 Oe and returning to 100 Oe, the resistance measurement results were obtained as squares and triangles respectively as shown in Fig. 6. In the case where the magnetic moments of both NiFe and LSMO are in parallel, when the magnetic field changes from 100 Oe to −5 Oe, the resistance remains unchanged. When the field is over −5.0 Oe, soft magnet NiFe starts to switch. Thus, resistance change is obvious due to anti-parallel alignment of magnetic moment between NiFe and LSMO. From Fig. 6, two facts about this multiferroic junction memory can be seen. The first fact is that, under the two different ferroelectric polarizations of BTO and the two different ferromagnetic polarizations of NiFe and LSMO, there are four resistance states across the junction. With the up and down ferroelectric polarizations, the relative resistance changes of the spin valve for the parallel (ON state) and anti-parallel (OFF state) magnetizations are 500 Ω and 15 Ω, respectively; i.e., the resistance change due to the TMR effect is +0.3% to −0.15%. While when the spin valve is in the ON state (LSMO and NiFe are in the parallel magnetization), the ratio of the two resistance states for the different ferroelectric polarizations is 16. As a consequence, with the combination of ferroelectric switching and small-coercive field switching magnetic tunnel junction, four resistance states are obtained with less energy consumption. It is worth noting that the low TMR-ratio measured by others groups may be due to two possible reasons, i.e. the formation of dead layer in the polar-discontinuity model^35^,^36^ or the electrodes not actually being spin-polarized as expected^37^,^38^.

Another fact is that during the 200 cycles test (called the endurance test), the resistances (read with 0.1 V) for the four different states are relatively stable, indicating the good endurance proprieties. The increase of resistance when increasing the number of cycles may be due to the heat dissipation causing overall temperature increase throughout the experiment due to the metallic behavior of LSMO.

In conclusion, multiferroic tunneling junction based on NiFe/BTO/LSMO has been grown by laser-MBE technique. Ferroelectric switching induced resistive change memory with OFF/ON ratio of 16 and 0.3% TMR effect have been achieved in this tunneling structure. Relatively much lower magnetic switching field less than 100 Oe and four-state memory characteristics have been demonstrated with very good endurance for such structure. These results are promising for high-density low-power consumption non-volatile memory. Further work is needed to make this memory structure workable at room temperature with miniature memory cells in a cross bar device.

## Methods

### Sample preparation

BTO/LSMO bilayers were grown by laser-molecular beam epitaxy (Laser-MBE) on (001) SrTiO3 (STO) single crystal substrates using a KrF excimer laser (λ = 248 nm), with a flunece of 200 mJ. This substrate was chosen to induce a large compressive strain in ultrathin films of BTO, which enhances the ferroelectric polarization^13^,^14^,^39^. Before deposition, the base pressure in the chamber is under a high vacuum in about 2.0 × 10^−5^Pa in order to reduce residential gas. A 60 nm-thick LSMO film was grown at a deposition temperature of 730 °C, an oxygen pressure of 15Pa and the laser pulse repetition rate is 2 Hz. Then about 5 nm-thick BTO film was grown under an oxygen pressure of 10Pa at 700 °C with a repetition of 1 Hz. After deposition, the sample was *in-situ* annealed at 700 °C under a high oxygen pressure of 0.1 atm (10 kPa) for 1 hour to reduce oxygen vacancies in the oxide films. Then, the sample was cooled down to room temperature in the same ambient.

### Sample characterization

The microstructure of the BTO/LSMO/STO was characterized by high-resolution transmission electron microscopy (JEM 2100F). Piezoresponse Force Microscopy was performed using Pt/Ti-coated silicon cantilevers to obtain the phase and amplitude images to evaluate the ferroelectric property of BTO. For transportation measurement, top electrodes of the size of 100 μm in diameter were defined by photolithography and lift-off of NiFe layers, which was grown by magnetron sputtering at room temperature and covered by a layer of gold to prevent oxidation. A layer of sputter-deposited Au is in contact with LSMO to overcome the Schottky junction. The junction memory cells were contacted by Aluminum wire and E-solder for measuring transport properties. Current-voltage (I-V) characteristics and resistance switching properties were measured by using a Keithley 6517a source meter and magnetic properties are introduced by using VSM.

## Additional Information

**How to cite this article**: Yau, H. M. *et al.* Low-field Switching Four-state Nonvolatile Memory Based on Multiferroic Tunnel Junctions. *Sci. Rep.* **5**, 12826; doi: 10.1038/srep12826 (2015).

## Supplementary Material

Supplementary Information
