# Supplementary material for: Low-field Switching Four-state Nonvolatile Memory Based on Multiferroic Tunnel Junctions
Source: Sci Rep. 2015 Aug 4;5:12826. doi: 10.1038/srep12826 (PMC4523833; doi:10.1038/srep12826)
Supplement: Supplementary Information [file srep12826-s1.pdf]

Low-field Switching Four-state Nonvolatile Memory Based on Multiferroic Tunnel Junctions

H.M. Yau[1], *Z.B. Yan*[2], *N. Y. Chan*[1], *K. Au*[1], *C.M. Wong*[1], *C.W.Leung*[1], *F.Y. Zhang*[3], *X.S. Gao*[3] *and J.Y. Dai* [*,1]

[1]Department of Applied Physics, The Hong Kong Polytechnic University, Hung Hom, Kowloon, Hong Kong, P. R. China

[2]Laboratory of Solid State Microstructures, Nanjing University, Nanjing 210093, China

[3]Institute for Advanced Materials and Laboratory of Quantum Engineering and Quantum Materials, South China Normal University, Guangzhou 510006, China

Acting as an electrode, electrical resistance of LSMO film was measured by varying temperature from 20K to 350K. Two LSMO films with different growth conditions, deposited under high vacuum and under oxygen pressure of 15Pa, were deposited and measured. The result of temperature-dependent resistance shows that the film deposited under high vacuum decreases with increasing temperature. The peak of transition point is below 100K and this LSMO film behaves semiconducting. Although the resistance is smaller at room temperature, the Curie temperature of the LSMO film deposited at insufficient oxygen is too low that Mn 3d spins become disordered, and the spin anisotropy disappears based on the result of Park's [1]. Thus, this film cannot be used as a spin-valve bottom electrode.

In another deposition condition, sufficient oxygen was supplied during LSMO deposition. The result shows in Figure 1 distinguishes the difference due to different level of oxygen supplying. Under 15Pa oxygen deposition, temperature-dependent resistance displays a metallic behavior that temperature

increases with resistance increases. Also, the Curie point is above 300K since no peak can be observed within 20K to 300K.

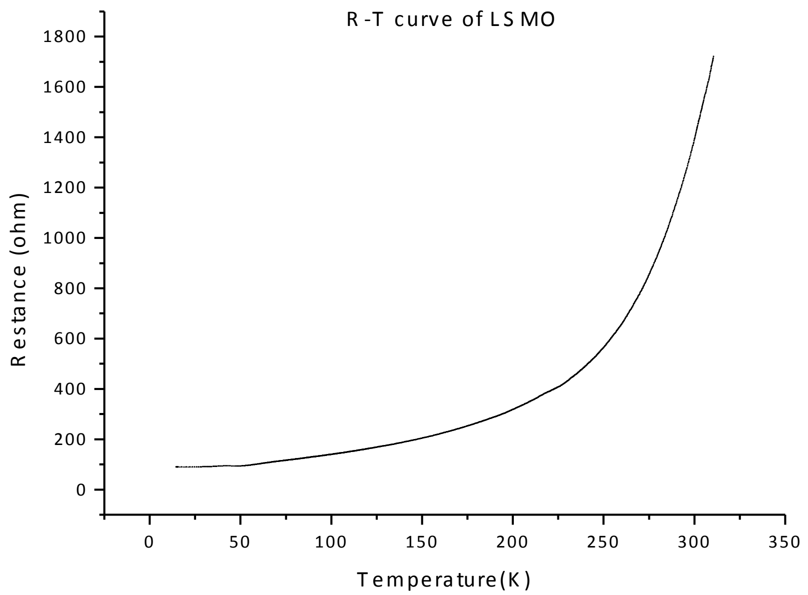

Figure S1. Resistance against temperature curve of $La_{0.7}Sr_{0.3}MnO_3$ deposited under 15Pa O_2 shows metallic behavior and Curie temperature is above room temperature.

Hole-doped manganese oxides $La_{1-x}Sr_xMnO_3$ with perovskite structure becomes conducting despite of the fact that its parent antiferromagnetic LaMnO3 is an insulator. Due to the deviation of e_g band filling process, $La_{1-x}Sr_xMnO_3$ undergoes an insulator-to-metal transition. A strong exchange interaction between e_g electrons or hole and t_{2g} spins leads to the distinct feature of metallic state in LSMO. It is shown in Figure S1, near the Tc point, the resistance is almost ~20 times magnitude higher than that in low temperature. This rapid decrease in resistance below Tc is explained by Tokura[2], and it is because of the increase of carrier mobility which results from reducing in the carrier scattering by thermal spin fluctuation. Based on double exchange theory [3], the ferromagnetic spin arrangement tends to increase the effective transfer interaction and thus the mobility of hole, finally results in drop of resistance right below Tc. When temperature is higher than Tc, semiconducting behavior is observed due to

paramagnetic spin configuration, localization effect of e_g hole-carriers will reduce the mobility significantly. As a consequence, to obtain a good performance of transport properties of LSMO, sufficient oxygen pressure is needed during deposition.

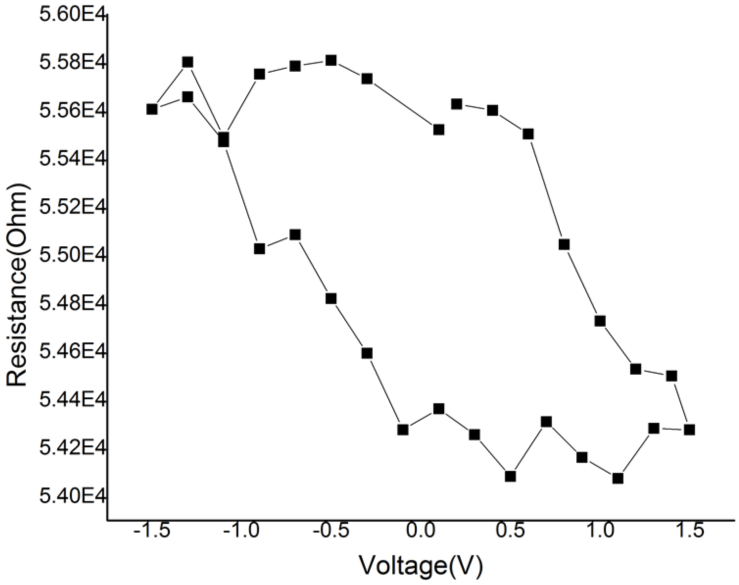

Figure S2. Resistance changes at room temperature

At first, to fabricate the FTJ that can be used in room temperature, testing for transport properties of the devices was carried out at room temperature. The result obtained in Figure S2 shows the resistance change at room temperature. The resistance changes from 54.1kΩ (low resistance state, LRS) to 55.8kΩ (high resistance state, HRS) with an extremely small OFF/ON ratio only. In O.Trithaveesak report[4], it was stated that the leakage currents can be observed when the BTO film thickness is reduced to 100nm. To solve this problem caused by thermally activated processes, measurement at lower temperature has to be carried out in order to reduce the conductivity in the system.

Due to the current-leakage problem of our device, a load resistance R_{load} acts as a current limiter after the resistance drop on the device in order to clarify the properties of resistance changing in BTO. From Figure S3(a), the ON/OFF ratio of BTO reaches 3 orders of magnitude by adding a constant resistance in

the measurement. It can be seen that the ratio is remaining constant after a thousand cycle endurance test as well as the retention test. Combining the TEM images and the properties of BTO below, it indicates the reason why resistance change is not very large because of current limitation of our equipment instead of the oxidation state of NiFe/BTO interface.

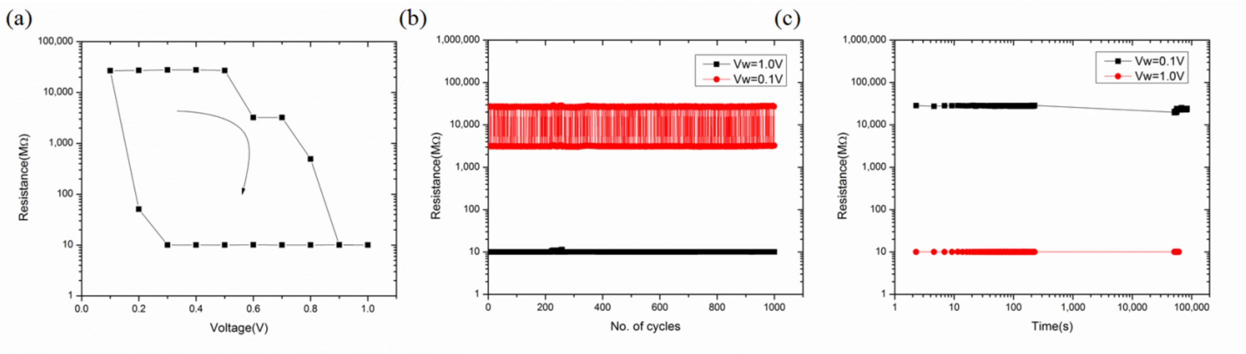

Figure S3. (a) Electroresistance change of NiFe/BTO/LSMO device; (b) and (c) are the tests for endurance in 1000cycles and retention respectively

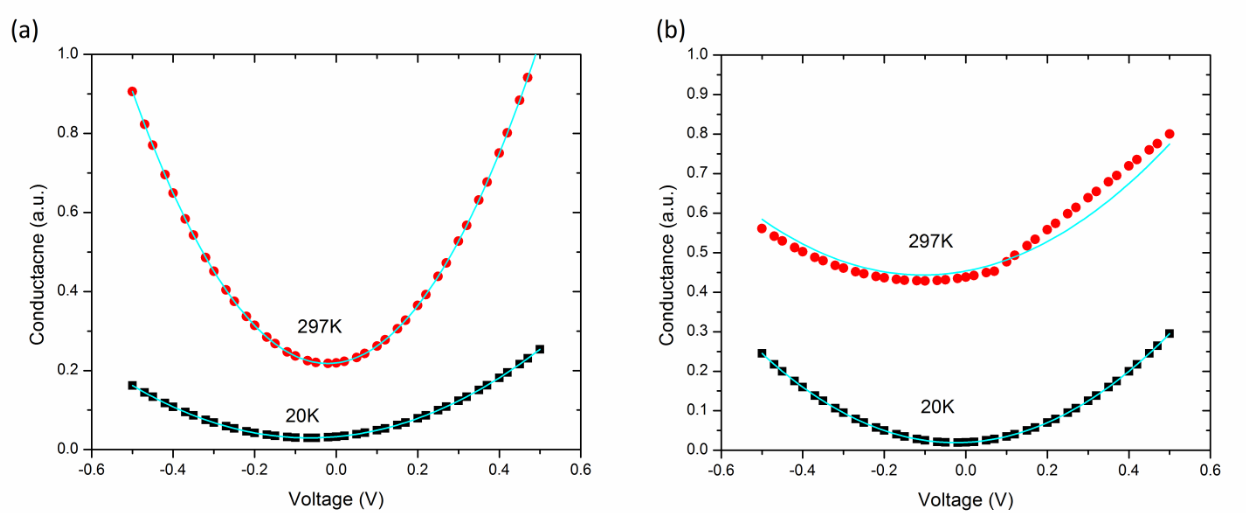

Figure S4. Tunneling properties of the device at 297K and 20K (a) ON state (b) OFF state

In order to determine whether the switching is due to a tunneling effect, the graph of conductance against voltage shows the direct tunneling mechanism. According to Brinkman's model, it suggests a trapezoidal barrier led to a roughly parabolic dependence of conductance on voltage [5].The expression

accurate to 10% when the barrier thickness is greater than 10Å and when $\frac{\Delta\varphi}{\bar{\varphi}}$ is less than one are using equations E1, E2 and E3,

$$\frac{G(V)}{G(0)} = 1 - \left(\frac{A_o \Delta\varphi}{16\bar{\varphi}^{\frac{1}{2}}}\right) eV + \left(\frac{9}{128}\frac{A_o{}^2}{\bar{\varphi}}\right)(eV)^2 \qquad \text{(E1)}$$

$$\mathrm{G}(0) = \frac{3.16\times10^{10}\bar{\varphi}^{\frac{1}{2}}}{d} exp\left(-1.025 d\bar{\varphi}^{\frac{1}{2}}\right) \qquad \text{(E2)}$$

$$A_o = \frac{4(2m)^{\frac{1}{2}} d}{3\hbar} \qquad \text{(E3)}$$

where $\Delta\varphi = \varphi_2 - \varphi_1$, difference of barrier heights on both interfaces of BTO/NiFe and BTO/LSMO with zero applied voltage, $\bar{\varphi}$ is the average barrier height, d is the barrier thickness in Å and the potentials are in volts. In our work, the LRS were fitted by this model and the solid line in Figure S4 indicates E1. It can be seen clearly that the curves in range of 500mV agree with Brinkman model which implies the electron tunneling is dominant in the transports process[6,7]. From the model, a table of parameters is obtained from the fitting.

State	Temperature(K)	d (nm)	$\bar{\varphi}$ (eV)	$\Delta\varphi$ (eV)
ON (LRS)	20	4.5	0.65	0.39
ON (LRS)	297	4.5	0.67	0.28
OFF (HRS)	20	4.5	0.88	0.41
OFF (HRS)	297	4.5	0.93	0.23

Table T1. Parameters obtained by Brinkman model.

In the obtained results in Table T1, the barrier heights at two interfaces (BTO/LSMO) and (NiFe/BTO) of both structure and at 20K are in agreement with the values recorded by the others, ranging from 0.5 to 1.1eV [8,9]. The asymmetric curves and a shift of the minimum value of conductance from zero voltage are caused by the different barrier heights at two electrode interfaces.

For the device measured in 20K, the value of the barrier thickness calculated is in the range of 10% difference. However, the difference is over 20% for the device that in 297K. It may be explained by two major reasons. Brinkman's model is derived at 0K, while 297K has a large difference in temperature, so other mechanisms may be involved. Another reason is that voltage-dependent hopping conductance may present at high temperature as reported by Oliver et al[10]. However, it is clear that the mechanism for transport is purely tunnel conduction caused by ferroelectric switching at 20K.

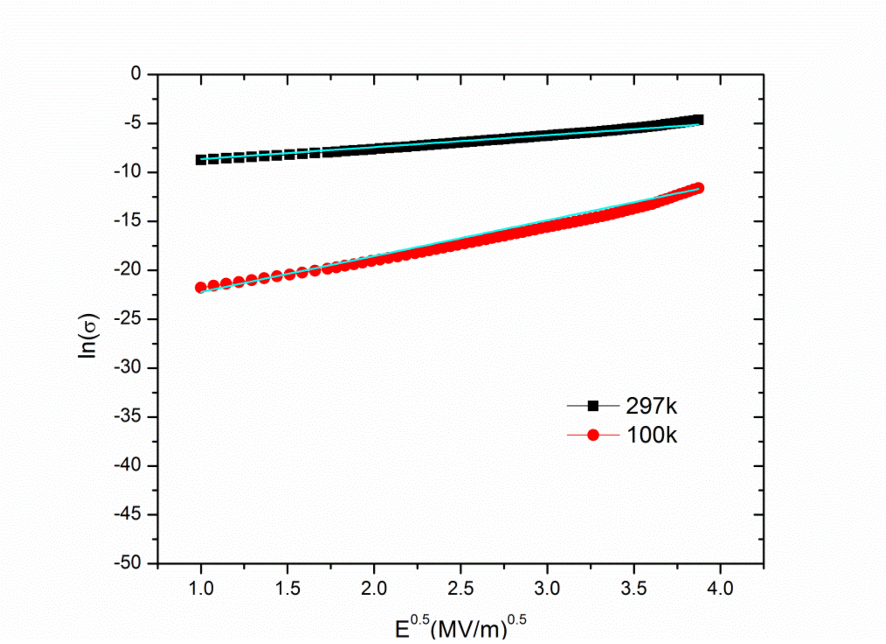

Figure S5. Tunneling model of the device at higher temperature

In 297K, the transport mechanisms of the device may include several according to the results above, to understand whether the switching consists of other transport mechanisms rather than tunneling effect in higher temperature, the graph ln(J/E) vs $E^{0.5}$ shows Poole-Frenkel conduction mechanism [11]. According to J.G.Simmons' model [11–13], it suggests the linearity at high voltage may because of the Poole-Frenkel (PF) or Schottky emission (SE). By using equation E4 and E5,

$$\sigma = \sigma_0 \exp\left(\frac{\beta E^{\frac{1}{2}}}{kT}\right) \qquad \text{(E4)}$$

$$\beta = \sqrt{\frac{e^3}{\alpha \pi \varepsilon_0 K}} \qquad \text{(E5)}$$

Where σ_0 is the low field conductivity of the system, k is the Boltzmann constant, β is the slope of the plot ln σ vs $E^{0.5}/kT$, ε_0 is the permittivity of free space and K is the high-frequency dielectric constant of the insulator. In case of Poole-Frenkel mechanism, α =1 while that of Schottky emission α =4. The resulting value for K should satisfy $K=n^2$, where n is the refractive index of the material. In our work, the value of K are 4.45 to 6.5 and 1.1 to 2 , respectively, for PF and SE. compared with the expected value of optical dielectric constant of BTO which is 2.4 [14,15], SE can be neglected and PF conducting mechanism would be dominated in higher temperature. SCLC can also be ignored because the result does not show a discrete value which is not shown here[16].

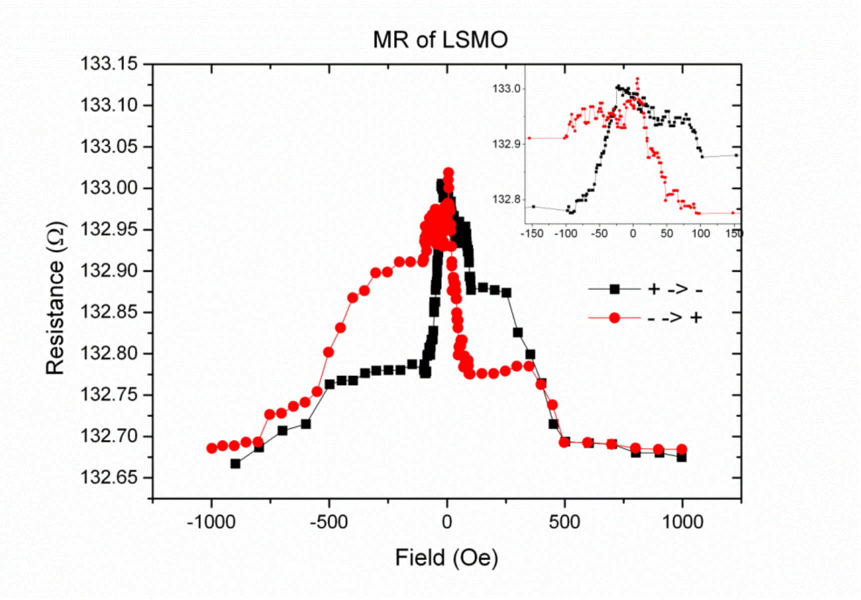

Figure S6. Resistance changes at 8K by varying the magnetic field

To clarify whether the involvement of colossal magnetoresistance or anisotropic magnetoresistance of LSMO may give rise to misinterpretations when comparing resistance with and without magnetic field,

i.e., the resistance change is not related to TMR, resistance of plain-LSMO was measured under the testing condition same as that of the device. The result is shown in Figure S6, where it can be seen that the change of resistance is only 0.275Ω for antiparallel and parallel magnetic moment, while the resistance difference of the device is much greater than plain layer of LSMO. Magnetoresistance of the NiFe/BTO/LSMO is hence TMR effect.

References:

1. Park, J.-H. *et al.* Direct evidence for a half-metallic ferromagnet. *Nature* **392,** 794–796 (1998).

2. Tokura, Y. *et al.* Giant Magnetotransport Phenomena in Filling-Controlled Kondo Lattice System: La1-xSrxMnO3. *J. Phys. Soc. Japan* **63,** 3931–3935 (1994).

3. Anderson, P. W. & Hasegawa, H. Considerations on double exchange. *Phys. Rev.* **100,** 675–681 (1955).

4. Trithaveesak, O., Schubert, J. & Buchal, C. Ferroelectric properties of epitaxial BaTiO[sub 3] thin films and heterostructures on different substrates. *J. Appl. Phys.* **98,** 114101 (2005).

5. Brinkman, W. F. Tunneling Conductance of Asymmetrical Barriers. *J. Appl. Phys.* **41,** 1915 (1970).

6. Pantel, D., Goetze, S., Hesse, D. & Alexe, M. Reversible electrical switching of spin polarization in multiferroic tunnel junctions. *Nat. Mater.* **11,** 289–93 (2012).

7. Chanthbouala, A. *et al.* Solid-state memories based on ferroelectric tunnel junctions. *Nat. Nanotechnol.* **7,** 101–4 (2012).

8. Wang, Z. *et al.* A physics-based compact model of ferroelectric tunnel junction for memory and logic design. *J. Phys. D. Appl. Phys.* **47,** 045001 (2014).

9. Barrionuevo, D. *et al.* Tunneling electroresistance in multiferroic heterostructures. *Nanotechnology* **25,** 495203 (2014).

10. Oliver, B. Temperature and bias dependence of dynamic conductance—low resistive magnetic tunnel junctions. *J. Appl. Phys.* **95,** 546 (2004).

11. Murari, N. M., Thomas, R., Pavunny, S. P., Calzada, J. R. & Katiyar, R. S. DyScO[sub 3] buffer layer for a performing metal-ferroelectric-insulator-semiconductor structure with multiferroic BiFeO[sub 3] thin film. *Appl. Phys. Lett.* **94,** 142907 (2009).

12. Simmons, J. Poole-Frenkel Effect and Schottky Effect in Metal-Insulator-Metal Systems. *Phys. Rev.* **155,** 657–660 (1967).

13. Specht, M., Städele, M., Jakschik, S. & Schröder, U. Transport mechanisms in atomic-layer-deposited Al[sub 2]O[sub 3] dielectrics. *Appl. Phys. Lett.* **84,** 3076 (2004).

14. W. N. Lawless. Anisotropic Oxygen Polarizability in BaTiO3. *Phys. Rev.* **138,** (1965).

15. P. W. Forsbergh, J. Domain Structures and Phase Transitions in Barium Titanate. *Phys. Rev.* **76,** 1187 (1949).

16. Wang, D. Y. *et al.* Ferroelectric, piezoelectric, and leakage current properties of (K[sub 0.48]Na[sub 0.48]Li[sub 0.04])(Nb[sub 0.775]Ta[sub 0.225])O[sub 3] thin films grown by pulsed laser deposition. *Appl. Phys. Lett.* **98,** 022902 (2011).
